# Radiomics-Based Machine Learning in Differentiation Between Glioblastoma and Metastatic Brain Tumors

**DOI:** 10.3389/fonc.2019.00806

**Published:** 2019-08-22

**Authors:** Chaoyue Chen, Xuejin Ou, Jian Wang, Wen Guo, Xuelei Ma

**Affiliations:** ^1^Department of Biotherapy, Cancer Center, West China Hospital, Sichuan University, Chengdu, China; ^2^State Key Laboratory of Biotherapy and Cancer Center, West China Hospital, Collaborative Innovation Center for Biotherapy, Sichuan University, Chengdu, China; ^3^Department of Neurosurgery, West China Hospital, Sichuan University, Chengdu, China; ^4^West China School of Medicine, West China Hospital, Sichuan University, Chengdu, China; ^5^School of Computer Science, Nanjing University of Science and Technology, Nanjing, China

**Keywords:** radiomics, machine learning, glioblastomas, metastatic brain tumors, texture analysis

## Abstract

**Purpose:** To investigative the diagnostic performance of radiomics-based machine learning in differentiating glioblastomas (GBM) from metastatic brain tumors (MBTs).

**Method:** The current study involved 134 patients diagnosed and treated in our institution between April 2014 and December 2018. Radiomics features were extracted from contrast-enhanced T1 weighted imaging (T1C). Thirty diagnostic models were built based on five selection methods and six classification algorithms. The sensitivity, specificity, accuracy, and area under curve (AUC) of each model were calculated, and based on these the optimal model was chosen.

**Result :** Two models represented promising diagnostic performance with AUC of 0.80. The first model was a combination of Distance Correlation as the selection method and Linear Discriminant Analysis (LDA) as the classification algorithm. In the training group, the sensitivity, specificity, accuracy, and AUC were 0.75, 0.85, 0.80, and 0.80, respectively; and in the testing group, the sensitivity, specificity, accuracy, and AUC of the model were 0.69, 0.86, 0.78, and 0.80, respectively. The second model was the Distance Correlation as the selection method and logistic regression (LR) as the classification algorithm, with sensitivity, specificity, accuracy, and AUC of 0.75, 0.85, 0.80, 0.80 in the training group and 0.69, 0.86, 0.78, 0.80 in the testing group.

**Conclusion:** Radiomic-based machine learning has potential to be utilized in differentiating GBM from MBTs.

## Introduction

Glioblastomas (GBM) and metastatic brain tumors (MBTs) are commonly identified brain tumors in the adult population. Pre-surgery diagnosis between these lesions is critical to assist in efficient treatment planning, especially for MBTs with brain metastases detected before the primary tumor (1). Magnetic resonance imaging (MRI) is highly recommended for radiological examination as a non-invasive tool due to the advantage of identifying the location and size of lesions (2, 3). However, conventional MR imaging is limited in differentiating GBM from solitary MBTs due to lacking characteristics on their imaging, and their contrast-enhancement patterns may mimic each other. Moreover, advanced MR techniques, like Dynamic Susceptibility Contrast Enhanced (DSC) MR imaging and proton magnetic resonance spectroscopy (HMRS), are not significant in the diagnosis of these lesions either given the similarities and the increased vascularity between these tumors or the metabolite ratios (4–8). Evidently, even with the quantitative information that individual MR techniques provided on specific properties of the tumor, the single radiological technique is not enough to provide a tumor characterization.

Considering MR data was able to reflect the pathophysiology of tumors visually, the quantitative radiomics-based analysis may provide a feasible solution to assist in the demanding process. Texture analysis (TA) is the mathematical method to calculate the voxel-intensity heterogeneity of images, including computed tomography (CT) and magnetic resonance imaging (MRI), and showed promising diagnostic ability in various lesions (9, 10). Previous studies have investigated the diagnostic ability of pattern recognition techniques combined with TA in order to aid physicians in making clinical decisions (3, 11, 12). However, the optimal diagnostic model is still controversial because the performance of models could be significantly different with various combinations of classification algorithms and the selection method on radiomics features. In the present study, we performed a radiomic-based machine learning method in discriminating GBM from MBTs with five selection methods and six classification algorithms to bring about the intuitional selection of an optimal model. Therefore, the purpose of our study was to assess the contribution of pattern recognition techniques using radiomics features in the different models to distinguish GBM from MBTs and to select the optimum one in terms of diagnostic value.

## Methods

### Patient and MR Imaging Sequence Selection

**Figure 1:**
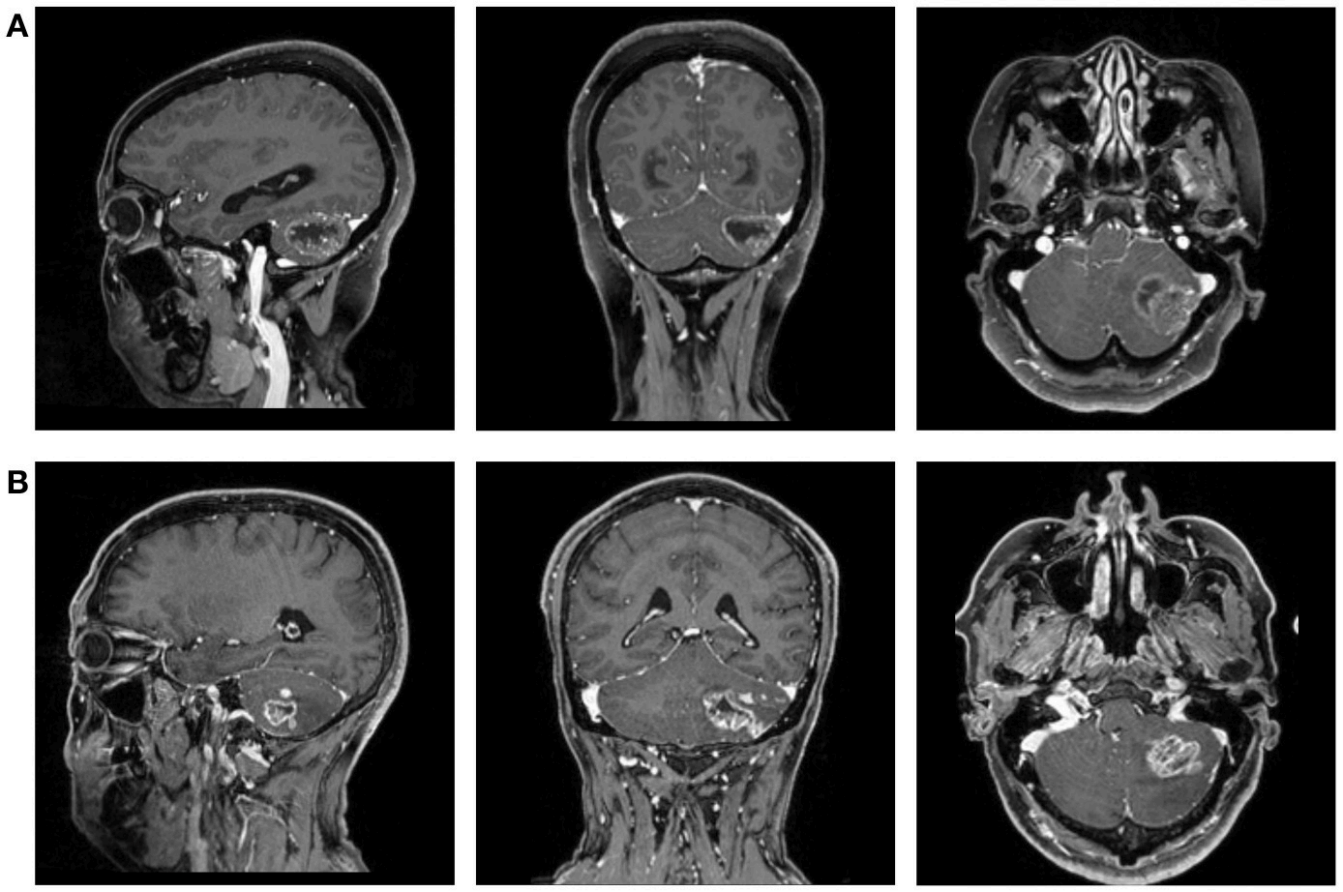
The magnetic resonance images (T1C) of a patient with **(A)** GBM and **(B)** MBTs.

This retrospective study was performed in our institution. The patients were selected from the neurosurgery department treated between April 2014 and December 2018. The initial selection enrolled potentially qualified patients who had records of intraoperative frozen-section confirmation on GBM or MBTs. Then we viewed the electronic medical records to collect the information we needed for analysis, including name, gender, age, and pathology report. Patients were excluded if the history of other types of intracranial diseases were documented or observed in MRI. The preoperative MR images were also collected from the radiological department through Picture Archiving and Communication Systems (PACS) (Figure 1).

In this study, we focused on conventional MR sequences, including T1-weighted imaging (T1WI), contrast-enhanced T1-weighted imaging (T1C), T2-weighted imaging (T2WI), and fluid attenuated inversion recovery (FLAIR), as they are the routine examination for patients with intracranial tumor. After the initial evaluation on images, contrast-enhanced T1 weighted-imaging (T1C) was chosen among all the sequences for further analysis due to the rather precise separation of tumor tissue from brain tissue.

### Conventional MR Imaging Examination Protocols

The MR scans were performed using the 3.0T Siemens Trio Scanners in the MR Research Center. High-resolution 3-dimensional T1-weighted images were collected using MPRAGE sequence. The parameters were as follows: TR/TE/TI = 1,900/2.26/900 ms, 176 axial slices with thickness = 1 mm, axial FOV = 25.6 × 25.6 cm^2^, Flip angle = 9°, and data matrix = 256 × 256. Dimeglumine (0.1 mmol/Kg) was the contrast agent for contrast-enhanced imaging, and multi-directional data of contrast-enhanced MRI were collected during the continuous interval time of 90–250 s.

### Texture Feature Extraction

**Figure 2:**
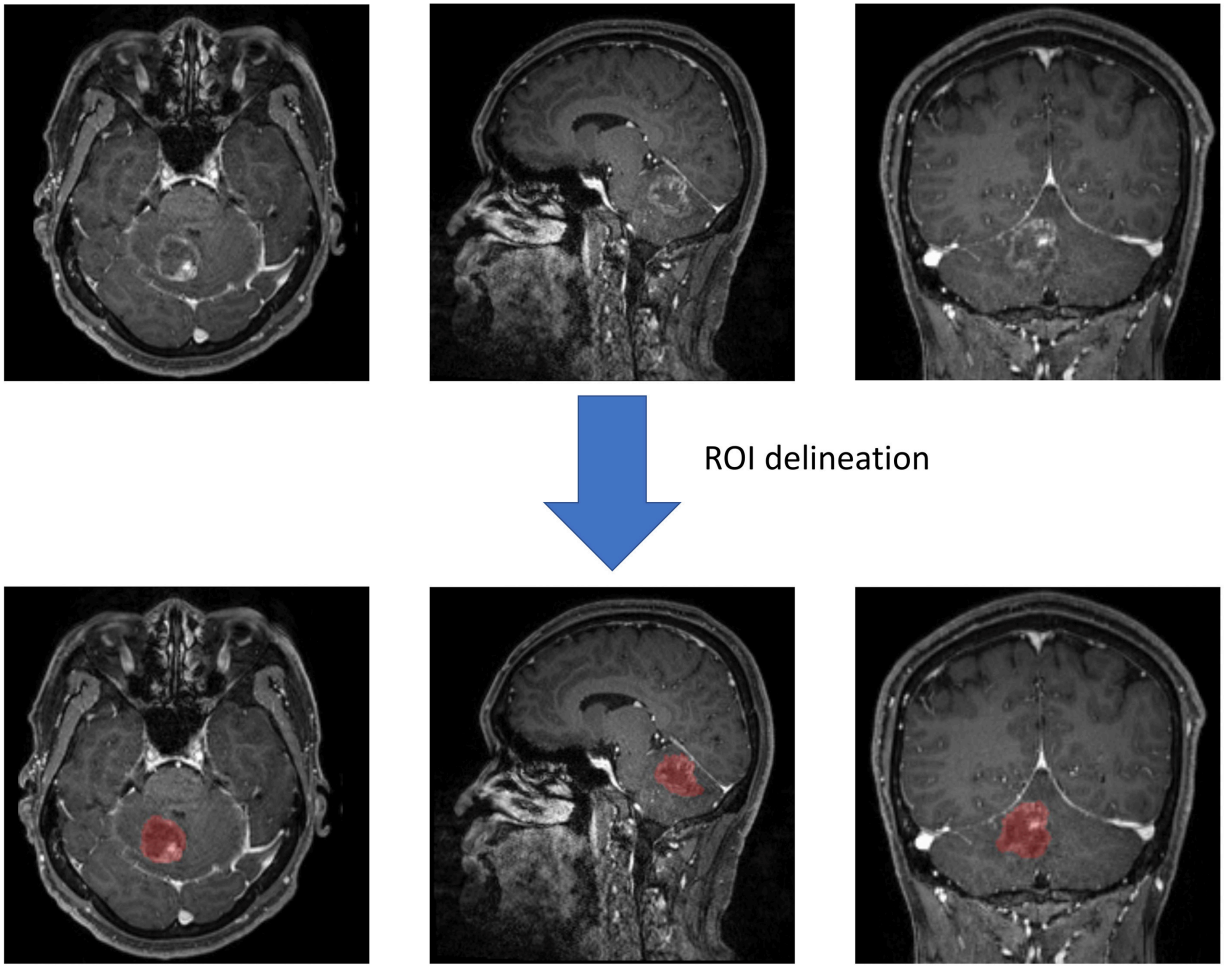
Screen capture of regions of interest (ROI) delineation.

Two neurosurgeons participated in the statistic extraction of texture features using LifeX software (http://www.lifexsoft.org) with the assistance of senior radiologists. Following the software protocol, they drew along the whole lesion in each slice to obtain the 3D-texture features. In each layer of the image, the regions of interest (ROI) were carefully drawn along the boundary of tumor tissue (including the necrosis and vessels within tissue). The peritumoral edema band and adjacent structure invasion were separated from the primary tumor with the difference in contrast enhancement. After segmentation on the whole tumor, the software automatically calculated and extracted texture features with default protocols (Figure 2). To ensure the validity and reproducibility of the procedure, the surgeons conducted data extraction twice, and the difference between two sets was examined with Manny-Whitney *U*-test. We adjusted the *q* < 0.01 as significant (before was *p* < 0.05) to avoid the interference of false-positive errors rising from a large number of texture features. The results suggested that none of the features were significantly different, implying that the results could be reliable and reproducible (Supplement Material 1).

Texture features were calculated from two orders. In the first order, features on shape- and histogram-based matrixes were extracted; and in the second order, features on the gray-level co-occurrence matrix (GLCM), neighborhood gray-level dependence matrix (NGLDM), gray-level zone length matrix (GLZLM), and gray-level run length matrix (GLRLM) were extracted. Finally, we built a statistical dataset of the radiomic statistics consisting of 43 features for machine-learning analysis.

### Classification Procedure

The establishment on the diagnostic model involved two parts: feature selection and classification algorithm (or known as classifier) deployment. The feature selection serviced the purpose that the numbers of features were so many that overfitting was inevitable for classification of algorithms. Considering the optimal selection method could be different for algorithms, five selection methods were evaluated in our study, including distance correlation, random forest (RF), least absolute shrinkage and selection operator (LASSO), eXtreme gradient boosting (Xgboost), and Gradient Boosting Decision Tree (GBDT). The selected features were adopted into classification algorithms to establish models.

Six classification algorithms were evaluated in our study, including Linear Discriminant Analysis (LDA, also known as Fisher Linear Discriminant), Support Vector Machine (SVM), random forest (RF), k-nearest neighbor (KNN), GaussianNB, and logistic regression (LR). Patients were divided as the training group and the testing group on a proportion of 4:1. Area under the receiver operating characteristic curve (AUC) of each model was calculated to assess their diagnostic performance. For each model, the progress of machine learning was repeated over 100 times to obtain the realistic distribution of classification accuracies.

All procedures involving human participants were in accordance with the ethical standards of the institutional and/or national research committee. The Ethics Committee of Sichuan University approved this retrospective study. The written informed consent was necessary before radiological examination (written informed consent for patients <16 years old was signed by parents or guardians) for all patients. They agreed to undertake examination if needed and were informed that the statistics (including MR image) might be used for academic purposes in the future.

## Result

### Patients Selection

A total number of 134 patients were enrolled in this study. Seventy-six of the patients were diagnosed with GBM, and 58 of them were diagnosed with MBTs. The average ages of patients were 46.9 and 57.6, respectively. The gender ratio for each type of tumor (Male: Female) was 10:9 and 9:5, respectively. The pathology reports represented that the majority of MBTs were originated from lung cancer and breast cancer (*N* = 54).

### Diagnostic Performance of Models

**Figure 3:**
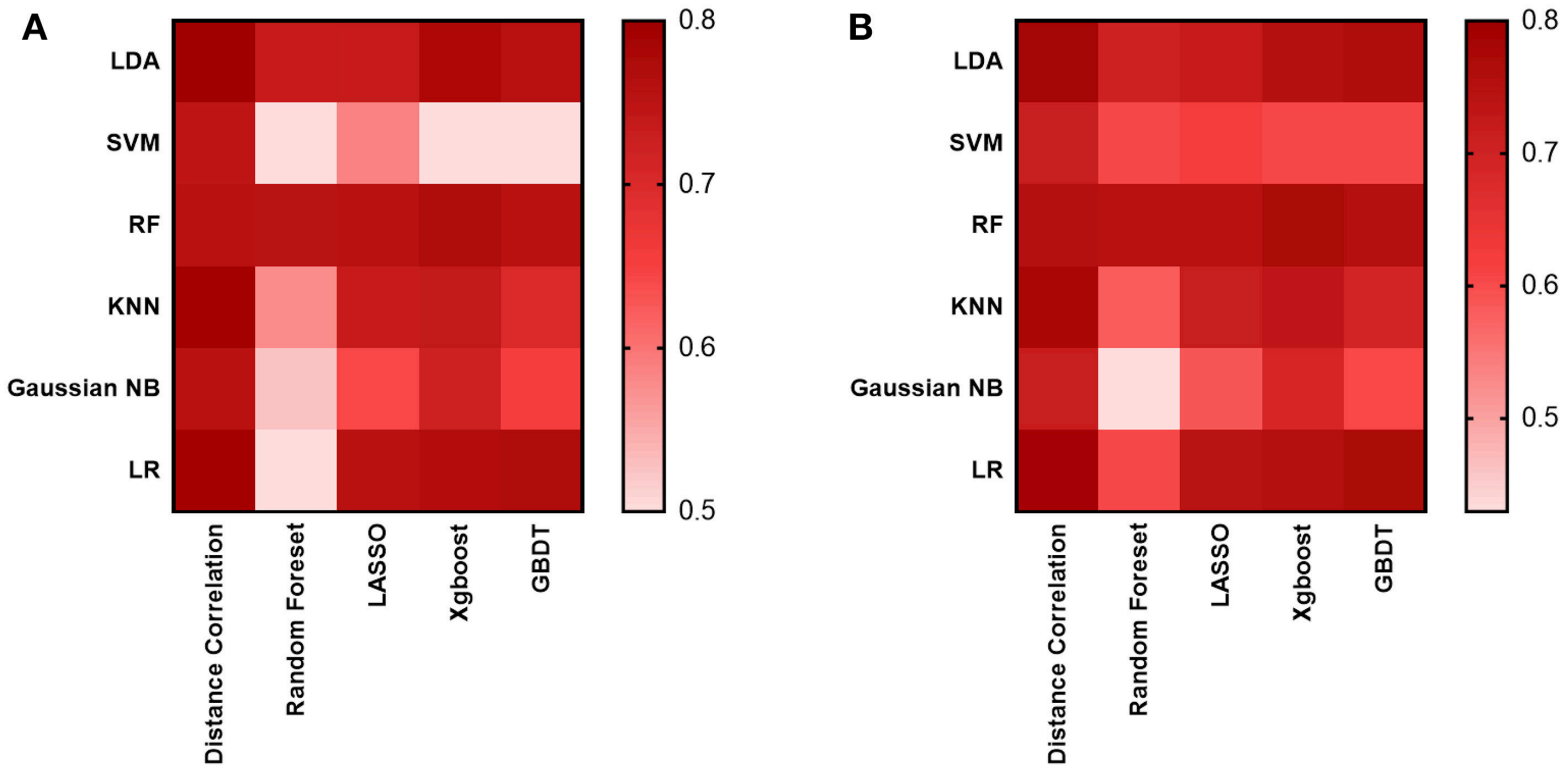
Heat map of the classifiers for differentiating between GBM and MBTs. **(A)** The AUC of the training group. **(B)** The AUC of the testing group.

As for the diagnostic models we evaluated, 30 models were established to select the suitable one, which was defined as the one with the highest AUC in the testing group. The results suggested the AUC of models mostly hovered around between 0.70 and 0.76 (Figure 3), and the highest value was 0.80 observed in two models: the Distance Correlation + LDA and the Distance Correlation + LR (Table 1). The details of each model performance are summarized in Supplement Material 2.

**Table 1:** Results of the optimal discriminative model in distinguishing GBM from MBTs in the training and the testing groups.

**Model**	**Training group**				**Testing group**			
	**AUC**	**Accuracy**	**Sensitivity**	**Specificity**	**AUC**	**Accuracy**	**Sensitivity**	**Specificity**
Distance correlation + LDA	0.80	0.80	0.75	0.85	0.80	0.78	0.69	0.86
Distance correlation + LR	0.83	0.83	0.79	0.87	0.80	0.79	0.71	0.85

*AUC, area under curve; LDA, linear discriminant analysis; LR, Logistic Regression*.

**Figure 4:**
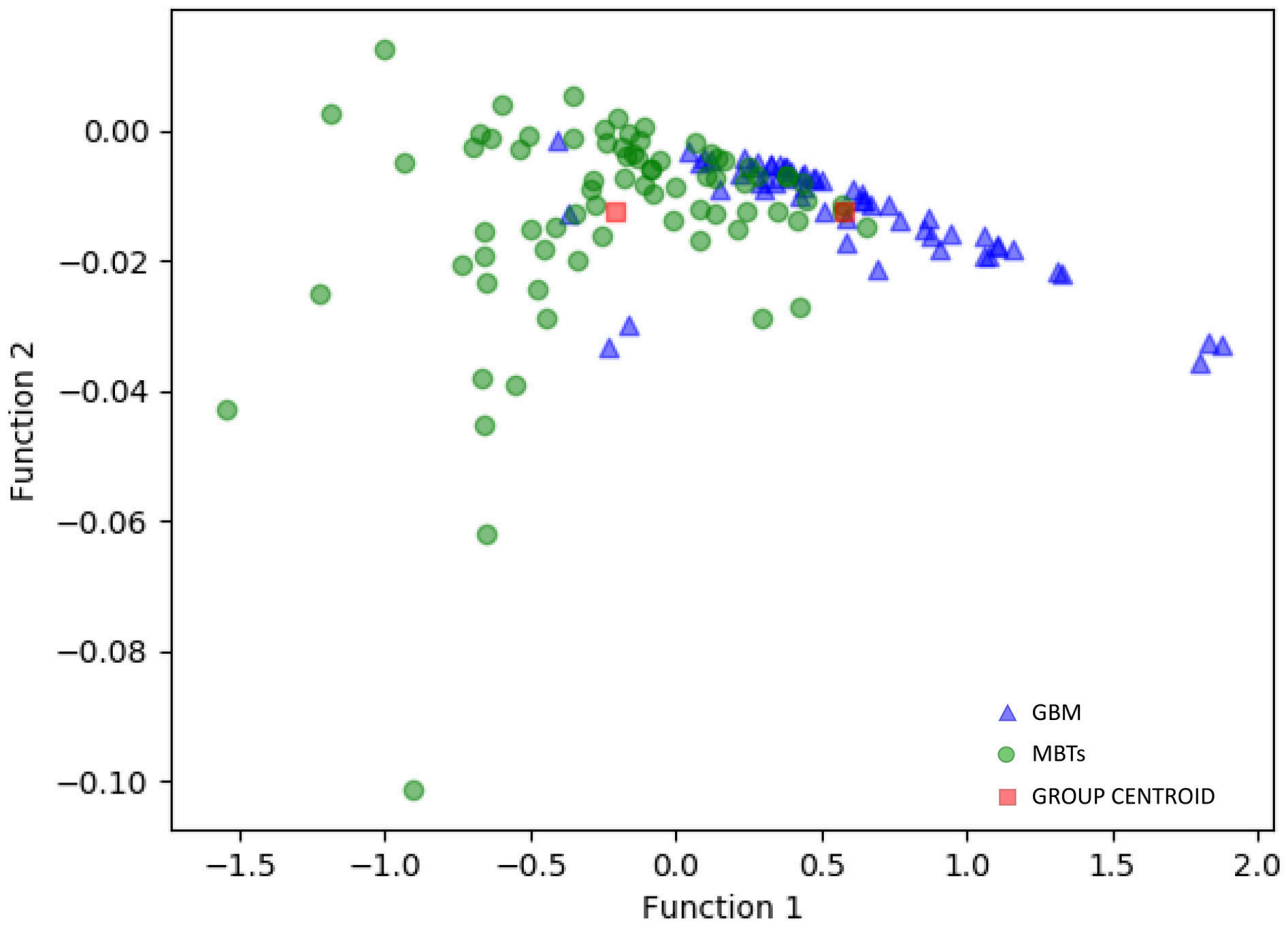
Distribution of the discriminant functions of LDA in discriminating GBM from MBTs.

**Figure 5:**
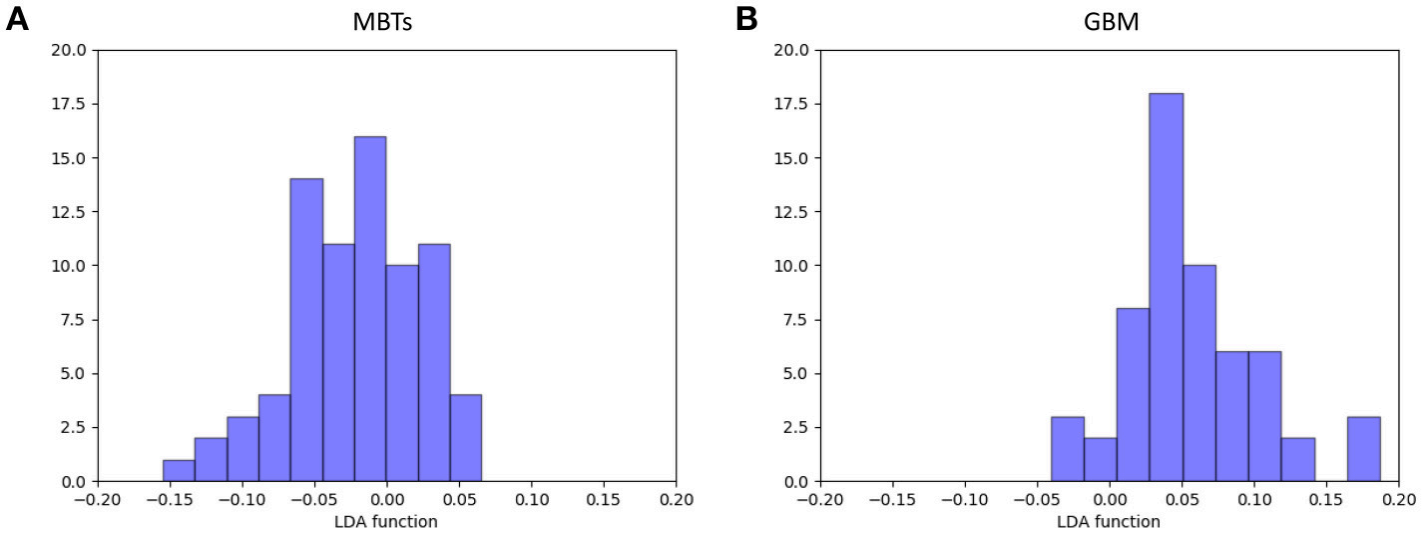
Example of distributions of the LDA function of **(A)** MBTs and **(B)** GBM for one cycle.

For the first model (the Distance Correlation + LDA), in the training group, the sensitivity, specificity, accuracy, and AUC of the model were 0.75, 0.85, 0.80, and 0.80, respectively. And in the testing group, the sensitivity, specificity, accuracy, and AUC of the model were 0.69, 0.86, 0.78, and 0.80. For the second model (the Distance Correlation + LR) in the training group, the sensitivity, specificity, accuracy, and AUC of the model were 0.79, 0.87, 0.83, and 0.83, respectively. And in the testing group, the sensitivity, specificity, accuracy, and AUC of the model were 0.71, 0.85, 0.79, and 0.80, respectively. The LDA distribution suggested these two models represented similar diagnostic performance (Figure 4). Figure 5 shows one example of 100 independent validation cycles of the model, representing the distribution of the first and second direct LDA canonical functions.

## Discussion

In the present study, we investigated the diagnostic ability of pattern recognition techniques combined with texture features extracted from conventional MRI in discriminating GBM from MBTs. MRI could provide excellent information on soft tissue differentiation to enable the exact localization of the tumors and to assist in the prediction of tumor response to therapy evaluation (13). However, pathological identification is the weakness of conventional MRI bringing additional advanced imaging techniques, which required additional fees and equipment, into tumor characterization and treatment. Our study made the evaluation on six classification algorithms consisting of five selection methods and six classification algorithms to identify the optimal model.

The diagnosis between MBTs and GBM on conventional MRI is rather straightforward because of the clinical history or observation of multiple lesions. The differences in tumor growth could lead to characteristic descriptions that GBM usually extends by infiltration, while MBTs usually arise within the brain parenchyma and grow by expansion, leading to comprising surrounding brain tissue (14). However, the emergence of lesions with a solitary enhancing appearance lacking information on primary tumors brings difficulty on differential diagnosis because high-grade GBM can present similar contrast enhancement patterns (15). The accurate and early diagnosis of these lesions is clinically important because the surgical planning, medical staging, and therapeutic approach can significantly vary from each other. Given that MR scan is the conventional radiological examination for patients, TA on T1C has the potential to serve as a feasible solution in clinical application without requiring additional fees. Previous studies have illustrated that TA combined with machine learning could assist in the diagnosis of various brain tumors, such as GBM from primary central nerve system lymphoma and meningioma from GBM (16, 17). Moreover, it has also been applied in tumor grade system and gene mutation prediction (18–22). The researchers illustrated the potential of artificial intelligence in lightening the clinical workload and improving early diagnostic accuracy.

Compared with the previous studies, our study involved various selection methods and classification algorithms to choose the optimal model with the best performance. Thirty models were evaluated, and two of them represented feasible diagnostic ability with AUC of 0.80 (the Distance Correlation + LDA and the Distance Correlation + LR). In the previous study, the SVM classifier was usually proven to be the suitable classifier compared to the others, which made sense considering that SVM is the suitable algorithm for small sample size. Our study illustrated that the feasible optimal classifiers were LDA and LR, while overfittings were almost observed in all SVM-based models (Supplement Material 2). LDA and LR are considered as the state-of-the-art on pattern recognition classifiers, with much better performance in some cases. LDA is also taken as the ground truth number of parameters in terms of performance. The mechanisms of classifiers provide a possible explanation of the differences in results. Both LDA and LR are the linear classifiers, while SVM is the non-linear classifier. The main difference of two types of classifiers consists in the shape of the decision boundary: plane or straight line in the first case, and surface or curved line in the second case. The choice of classification algorithm should be a tradeoff between computational burden and performance (23). This theory also demonstrated why SVM could be the suitable algorithm for a small sample size (50~60) while LDA/LR was suitable for a relatively large sample size (>100). However, it is worth noting that the diagnostic performances of classifiers did not improve much in the current research, even with the change in classification algorithm. All studies applying machine learning in discrimination of MBTs from GBM represented similar diagnostic performance with AUC in the testing group of ~0.80, even when radiomics features were selected with various selection methods and extracted from various sequences (11, 12, 24). More research is required to verify our results and to investigate the algorithm with better diagnostic performance.

There were some limitations in the current study. First and foremost, this study was a single central, retrospective study, bringing inevitable selection bias (Supplement Material 3). Second, the inhomogeneous histological subcategories of MBTs could reduce the accuracy in the differentiation. Future investigations with a larger sample size are required to assess the ability of classification algorithms and texture parameters in characterizing the lesion subtype. Third, only texture features retrieved from T1C images were adapted into classifiers, while features from other sequences (like T2WI and FLAIR) and advanced MR techniques were not explored. Fourth, the models were not validated in the other dataset, and we cannot guarantee the diagnostic ability of our models for external datasets due to the various protocols of imaging acquisition and MR scanners. However, the analysis protocol and image processing procedure were open-source packages and they should be validated and reproduced.

## Data Availability

The raw data supporting the conclusions of this manuscript will be made available by the authors, without undue reservation, to any qualified researcher.

## Author Contributions

XM participated in the conceptualization and revised intellectual content in the manuscript. CC collected MR image, participated in MRI features extraction, and drafted this manuscript. XO collected MR image, participated in MRI features extraction. JW deployed the machine-learning algorism and responsible for statistical analysis. WG assisted in MRI features extraction.

### Conflict of Interest Statement

The authors declare that the research was conducted in the absence of any commercial or financial relationships that could be construed as a potential conflict of interest.

## Supplementary Material

Supplement Material 1 The repeatability of texture feature extraction examined by Mann-Whitney *U*-test.

Supplement Material 2 The diagnostic performance of each model.

Supplement Material 3 The radiomics quality score of research.
