# Supplementary material for: Radiomics-Based Machine Learning in Differentiation Between Glioblastoma and Metastatic Brain Tumors
Source: Front Oncol. 2019 Aug 22;9:806. doi: 10.3389/fonc.2019.00806 (PMC6714109; doi:10.3389/fonc.2019.00806)
Supplement: Supplement Material 1 — The repeatability of texture feature extraction examined by Mann-Whitney U-test. [file Data_Sheet_1.PDF]

Hypothesis Test Summary

	Null Hypothesis	Test	Sig.	Decision
1	The distribution of minValue is the same across categories of First=1, Second=2.	Independent-Samples Mann-Whitney U Test	.200	Retain the null hypothesis.
2	The distribution of meanValue is the same across categories of First=1,Second=2.	Independent-Samples Mann-Whitney U Test	.397	Retain the null hypothesis.
3	The distribution of stdValue is the same across categories of First=1, Second=2.	Independent-Samples Mann-Whitney U Test	.116	Retain the null hypothesis.
4	The distribution of maxValue is the same across categories of First=1, Second=2.	Independent-Samples Mann-Whitney U Test	.174	Retain the null hypothesis.
5	The distribution of HISTO_Skewness is the same across categories of First=1, Second=2.	Independent-Samples Mann-Whitney U Test	.143	Retain the null hypothesis.
6	The distribution of HISTO_Kurtosis is the same across categories of First=1,Second=2.	Independent-Samples Mann-Whitney U Test	.547	Retain the null hypothesis.
7	The distribution of HISTO_Entropy_log10 is the same across categories of First=1, Second=2.	Independent-Samples Mann-Whitney U Test	.085	Retain the null hypothesis.
8	The distribution of HISTO_Energy is the same across categories of First=1,Second=2.	Independent-Samples Mann-Whitney U Test	.198	Retain the null hypothesis.

Asymptotic significances are displayed. The significance level is .05.

(continued)

Hypothesis Test Summary

	Null Hypothesis	Test	Sig.	Decision
**9**	The distribution of SHAPE_Volume (mL) is the same across categories of First=1,Second=2.	Independent-Samples Mann-Whitney U Test	.052	Retain the null hypothesis.
**10**	The distribution of SHAPE_Volume (# vx) is the same across categories of First=1,Second=2.	Independent-Samples Mann-Whitney U Test	.060	Retain the null hypothesis.
**11**	The distribution of SHAPE_Sphericity (only for 3D ROI (nZ>1) is the same across categories of First=1,Second=2.	Independent-Samples Mann-Whitney U Test	.839	Retain the null hypothesis.
**12**	The distribution of SHAPE_Compacity only for 3D ROI (nZ>1) is the same across categories of First=1,Second=2.	Independent-Samples Mann-Whitney U Test	.174	Retain the null hypothesis.
**13**	The distribution of GLCM_Homogeneity is the same across categories of First=1, Second=2.	Independent-Samples Mann-Whitney U Test	.120	Retain the null hypothesis.
**14**	The distribution of GLCM_Energy is the same across categories of First=1,Second=2.	Independent-Samples Mann-Whitney U Test	.228	Retain the null hypothesis.
**15**	The distribution of GLCM_Contrast is the same across categories of First=1,Second=2.	Independent-Samples Mann-Whitney U Test	.243	Retain the null hypothesis.

Asymptotic significances are displayed. The significance level is .05.

(continued)

Hypothesis Test Summary

	Null Hypothesis	Test	Sig.	Decision
**16**	The distribution of GLCM_Correlation is the same across categories of First=1, Second=2.	Independent-Samples Mann-Whitney U Test	.172	Retain the null hypothesis.
**17**	The distribution of GLCM_Entropy_log10 is the same across categories of First=1, Second=2.	Independent-Samples Mann-Whitney U Test	.191	Retain the null hypothesis.
**18**	The distribution of GLCM_Dissimilarity is the same across categories of First=1, Second=2.	Independent-Samples Mann-Whitney U Test	.191	Retain the null hypothesis.
**19**	The distribution of GLRLM_SRE is the same across categories of First=1,Second=2.	Independent-Samples Mann-Whitney U Test	.113	Retain the null hypothesis.
**20**	The distribution of GLRLM_LRE is the same across categories of First=1,Second=2.	Independent-Samples Mann-Whitney U Test	.201	Retain the null hypothesis.
**21**	The distribution of GLRLM_LGRE is the same across categories of First=1,Second=2.	Independent-Samples Mann-Whitney U Test	.448	Retain the null hypothesis.
**22**	The distribution of GLRLM_HGRE is the same across categories of First=1,Second=2.	Independent-Samples Mann-Whitney U Test	.409	Retain the null hypothesis.

Asymptotic significances are displayed. The significance level is .05.

(continued)

Hypothesis Test Summary

	Null Hypothesis	Test	Sig.	Decision
**23**	The distribution of GLRLM_SRLGE is the same across categories of First=1,Second=2.	Independent-Samples Mann-Whitney U Test	.486	Retain the null hypothesis.
**24**	The distribution of GLRLM_SRHGE is the same across categories of First=1, Second=2.	Independent-Samples Mann-Whitney U Test	.433	Retain the null hypothesis.
**25**	The distribution of GLRLM_LRLGE is the same across categories of First=1,Second=2.	Independent-Samples Mann-Whitney U Test	.406	Retain the null hypothesis.
**26**	The distribution of GLRLM_LRHGE is the same across categories of First=1, Second=2.	Independent-Samples Mann-Whitney U Test	.876	Retain the null hypothesis.
**27**	The distribution of GLRLM_GLNU is the same across categories of First=1,Second=2.	Independent-Samples Mann-Whitney U Test	.083	Retain the null hypothesis.
**28**	The distribution of GLRLM_RLNU is the same across categories of First=1,Second=2.	Independent-Samples Mann-Whitney U Test	.127	Retain the null hypothesis.
**29**	The distribution of GLRLM_RP is the same across categories of First=1,Second=2.	Independent-Samples Mann-Whitney U Test	.150	Retain the null hypothesis.

Asymptotic significances are displayed. The significance level is .05.

(continued)

Hypothesis Test Summary

	Null Hypothesis	Test	Sig.	Decision
**30**	The distribution of NGLDM_Coarseness is the same across categories of First=1, Second=2.	Independent-Samples Mann-Whitney U Test	.087	Retain the null hypothesis.
**31**	The distribution of NGLDM_Contrast is the same across categories of First=1, Second=2.	Independent-Samples Mann-Whitney U Test	.421	Retain the null hypothesis.
**32**	The distribution of NGLDM_Busyness is the same across categories of First=1, Second=2.	Independent-Samples Mann-Whitney U Test	.301	Retain the null hypothesis.
**33**	The distribution of GLZLM_SZE is the same across categories of First=1,Second=2.	Independent-Samples Mann-Whitney U Test	.205	Retain the null hypothesis.
**34**	The distribution of GLZLM_LZE is the same across categories of First=1,Second=2.	Independent-Samples Mann-Whitney U Test	.155	Retain the null hypothesis.
**35**	The distribution of GLZLM_LGZE is the same across categories of First=1,Second=2.	Independent-Samples Mann-Whitney U Test	.344	Retain the null hypothesis.
**36**	The distribution of GLZLM_HGZE is the same across categories of First=1,Second=2.	Independent-Samples Mann-Whitney U Test	.304	Retain the null hypothesis.

Asymptotic significances are displayed. The significance level is .05.

(continued)

Hypothesis Test Summary

	Null Hypothesis	Test	Sig.	Decision
**37**	The distribution of GLZLM_SZLGE is the same across categories of First=1,Second=2.	Independent-Samples Mann-Whitney U Test	.909	Retain the null hypothesis.
**38**	The distribution of GLZLM_SZHGE is the same across categories of First=1,Second=2.	Independent-Samples Mann-Whitney U Test	.264	Retain the null hypothesis.
**39**	The distribution of GLZLM_LZLGE is the same across categories of First=1,Second=2.	Independent-Samples Mann-Whitney U Test	.152	Retain the null hypothesis.
**40**	The distribution of GLZLM_LZHGE is the same across categories of First=1,Second=2.	Independent-Samples Mann-Whitney U Test	.266	Retain the null hypothesis.
**41**	The distribution of GLZLM_GLNU is the same across categories of First=1,Second=2.	Independent-Samples Mann-Whitney U Test	.132	Retain the null hypothesis.
**42**	The distribution of GLZLM_ZLNU is the same across categories of First=1,Second=2.	Independent-Samples Mann-Whitney U Test	.697	Retain the null hypothesis.
**43**	The distribution of GLZLM_ZP is the same across categories of First=1,Second=2.	Independent-Samples Mann-Whitney U Test	.122	Retain the null hypothesis.

Asymptotic significances are displayed. The significance level is .05.
