# Supplementary material for: Radiomics-Based Machine Learning in Differentiation Between Glioblastoma and Metastatic Brain Tumors
Source: Front Oncol. 2019 Aug 22;9:806. doi: 10.3389/fonc.2019.00806 (PMC6714109; doi:10.3389/fonc.2019.00806)
Supplement: Supplement Material 2 — The diagnostic performance of each model. [file Data_Sheet_2.PDF]

Supplement material 2 The diagnostic performance of each model

Classifier			Distance Correlation	Random Forest	Lasso	Xgboost	GBDT
LDA	Training Group	Sensitivity	0.75	0.71	0.80	0.76	0.82
		Specificity	0.85	0.88	0.86	0.88	0.86
		accuracy	0.80	0.79	0.83	0.82	0.84
		AUC	0.80	0.80	0.83	0.83	0.84
	Testing Group	Sensitivity	0.69	0.59	0.62	0.66	0.70
		Specificity	0.86	0.83	0.81	0.84	0.80
		accuracy	0.78	0.70	0.72	0.75	0.76
		AUC	0.80	0.73	0.74	0.77	0.76
SVM	Training Group	Sensitivity	0.72	*1.00*	0.73	*1.00*	*1.00*
		Specificity	0.86	*1.00*	0.63	*1.00*	*1.00*
		accuracy	0.79	*1.00*	0.65	*1.00*	*1.00*
		AUC	0.79	*1.00*	0.61	*1.00*	*1.00*
	Testing Group	Sensitivity	0.61	*0.61*	0.55	*0.55*	*0.55*
		Specificity	0.81	*0.81*	0.64	*0.64*	*0.64*
		accuracy	0.71	*0.60*	0.62	*0.60*	*0.60*
		AUC	0.74	*0.50*	0.59	*0.50*	*0.50*
RF	Training Group	Sensitivity	0.88	0.88	0.90	0.92	0.90
		Specificity	0.85	0.85	0.85	0.85	0.84
		accuracy	0.86	0.86	0.87	0.88	0.86
		AUC	0.85	0.86	0.86	0.87	0.85
	Testing Group	Sensitivity	0.68	0.68	0.67	0.71	0.70
		Specificity	0.80	0.78	0.79	0.81	0.79
		accuracy	0.75	0.74	0.74	0.77	0.76
		AUC	0.75	0.75	0.76	0.77	0.76
KNN	Training Group	Sensitivity	0.82	0.74	0.80	0.79	0.75
		Specificity	0.89	0.82	0.88	0.86	0.85
		accuracy	0.86	0.79	0.84	0.83	0.80
		AUC	0.86	0.79	0.84	0.83	0.80
	Testing Group	Sensitivity	0.68	0.48	0.62	0.64	0.60
		Specificity	0.85	0.68	0.80	0.80	0.76
		accuracy	0.77	0.58	0.71	0.73	0.69
		AUC	0.79	0.58	0.73	0.74	0.70
GaussianNB	Training Group	Sensitivity	0.65	0.46	0.55	0.63	0.57
		Specificity	0.94	0.80	0.90	0.91	0.88
		accuracy	0.76	0.48	0.63	0.73	0.66
		AUC	0.78	0.53	0.66	0.75	0.69
	Testing Group	Sensitivity	0.59	0.41	0.49	0.57	0.50
		Specificity	0.92	0.85	0.87	0.88	0.86
		accuracy	0.71	0.43	0.59	0.69	0.61
		AUC	0.75	0.53	0.64	0.72	0.65
LR	Training Group	Sensitivity	0.79	0.79	0.83	0.77	0.81
		Specificity	0.87	0.87	0.82	0.86	0.82
		accuracy	0.83	0.56	0.82	0.81	0.82
		AUC	0.83	0.50	0.81	0.82	0.81

Testing Group	Sensitivity	0.71	0.71	0.68	0.67	0.71
	Specificity	0.85	0.85	0.78	0.83	0.81
	accuracy	0.79	0.60	0.74	0.76	0.77
	AUC	0.80	0.50	0.76	0.77	0.77

Abbreviations:

LASSO: least absolute shrinkage and selection operator, Xgboost: eXtreme gradient boosting, GBDT: Gradient Boosting Decision Tree, LDA: linear discriminant analysis, SVM: support vector machine, RF: Random Forest, KNN: k-Nearest Neighbor, LR: Logistic Regression, AUC: area under curve
