# Supplementary material for: Radiomics-Based Machine Learning in Differentiation Between Glioblastoma and Metastatic Brain Tumors
Source: Front Oncol. 2019 Aug 22;9:806. doi: 10.3389/fonc.2019.00806 (PMC6714109; doi:10.3389/fonc.2019.00806)
Supplement: Supplement Material 3 — The radiomics quality score of research. [file Data_Sheet_3.PDF]

Supplement Material 3: The radiomics quality score of the research.

Criteria		Points	Scores
1	Image protocol quality - well-documented image protocols (for example, contrast, slice thickness, energy, etc.) and/or usage of public image protocols allow reproducibility/replicability	*+ 1* (if protocols are well-documented)<br>*+ 1* (if public protocol is used)	+ 2
2	Multiple segmentations - possible actions are: segmentation by different physicians/algorithms/software, perturbing segmentations by (random) noise, segmentation at different breathing cycles. Analyse feature robustness to segmentation variabilities	*+ 1*	+ 1
3	Phantom study on all scanners - detect inter-scanner differences and vendor-dependent features. Analyse feature robustness to these sources of variability	+ 1	0
4	Imaging at multiple time points - collect images of individuals at additional time points. Analyse feature robustness to temporal variabilities (for example, organ movement, organ expansion/ shrinkage)	*+ 1*	+ 1
5	Feature reduction or adjustment for multiple testing - decreases the risk of overfitting. Overfitting is inevitable if the number of features exceeds the number of samples. Consider feature robustness when selecting features	- 3 (if neither measure is implemented)<br>*+ 3* (if either measure is implemented)	+ 3
6	Multivariable analysis with non radiomics features (for example, EGFR mutation) - is expected to provide a more holistic model. Permits correlating/inferencing between radiomics and non radiomics features	*+ 1*	+ 1
7	Detect and discuss biological correlates - demonstration of phenotypic differences (possibly associated with underlying gene–protein expression patterns) deepens understanding of radiomics and biology	*+ 1*	+ 1
8	Cut-off analyses - determine risk groups by either the median, a previously published cut-off or report a continuous risk variable. Reduces the risk of reporting overly optimistic results	+ 1	0

9	Discrimination statistics - report discrimination statistics (for example, C-statistic, ROC curve, AUC) and their statistical significance (for example, p-values, confidence intervals). One can also apply resampling method (for example, bootstrapping, cross-validation)	*+ 1* (if a discrimination statistic and its statistical significance are reported)<br>*+ 1* (if a resampling method technique is also applied)	+ 2
10	Calibration statistics - report calibration statistics (for example, Calibration-in-the-large/slope, calibration plots) and their statistical significance (for example, *P*-values, confidence intervals). One can also apply resampling method (for example, bootstrapping, cross-validation)	*+ 1* (if a calibration statistic and its statistical significance are reported)<br>*+ 1* (if a resampling method technique is also applied)	+ 2
11	Prospective study registered in a trial database - provides the highest level of evidence supporting the clinical validity and usefulness of the radiomics biomarker	+ 7 (for prospective validation of a radiomics signature in an appropriate trial)	0
12	Validation - the validation is performed without retraining and without adaptation of the cut-off value, provides crucial information with regard to credible clinical performance	- 5 (if validation is missing)<br>*+ 2* (if validation is based on a dataset from the same institute)<br>+ 3 (if validation is based on a dataset from another institute)<br>+ 4 (if validation is based on two datasets from two distinct institutes)<br>+ 4 (if the study validates a previously published signature)<br>+ 5 (if validation is based on three or more datasets from distinct institutes)<br>*Datasets should be of comparable size and should have at least 10 events per model feature	+ 2
13	Comparison to 'gold standard' - assess the extent to which the model agrees with/is superior to the current 'gold standard' method (for example, TNM-staging for survival prediction). This comparison shows the added value of radiomics	*+ 2*	+ 2
14	Potential clinical utility - report on the current and potential application of the model in a clinical setting (for example, decision curve analysis).	*+ 2*	+ 2

15	Cost-effectiveness analysis - report on the cost-effectiveness of the clinical application (for example, QALYs generated)	*+ 1*	+ 1
16	Open science and data - make code and data publicly available. Open science facilitates knowledge transfer and reproducibility of the study	*+ 1* (if scans are open source)<br>*+ 1* (if region of interest segmentations are open source)<br>*+ 1* (if code is open source)<br>*+ 1* (if radiomics features are calculated on a set of representative ROIs and the calculated features and representative ROIs are open source)	+ 4
Total points (36 = 100%)			24
